# A simple nomogram for early postoperative risk prediction of clinically relevant pancreatic fistula after pancreatoduodenectomy

**DOI:** 10.1007/s00423-021-02184-y

**Published:** 2021-05-19

**Authors:** K. C. Honselmann, C. Antoine, L. Frohneberg, S. Deichmann, L. Bolm, R. Braun, H. Lapshyn, E. Petrova, T. Keck, U. Wellner, D. Bausch

**Affiliations:** 1 Department of Surgery, University Cancer Center, University Medical Center Schleswig-Holstein, Campus Luebeck, Luebeck, Germany; 2 Department of Surgery, Marien Hospital Herne-University Medical Center of the Ruhr-Universität Bochum, Hölkeskampring 40, 44625 Herne, Germany

**Keywords:** Pancreatic surgery, Pancreatic fistula, Risk prediction, POPF

## Abstract

**Purpose:**

Postoperative pancreatic fistulae (POPF) present a serious and life-threatening complication after pancreatic head resections (PD). Therefore, reliable risk stratification to identify those at risk is urgently needed. The aim of this study was to identify postoperative laboratory parameters for the prediction of POPF in the early postoperative period.

**Methods:**

One hundred eighty-two patients who underwent PD from 2012 until 2017 were retrospectively analyzed. Multivariate logistic regression was performed using the GLM (general linear model) method for model building. Two nomograms were created based on the GLM models of postoperative day one and postoperative day one to five. A cohort of 48 patients operated between 2018 and 2019 served as internal validation.

**Results:**

Clinically relevant pancreatic fistulae (CR-POPF) were present in 16% (*n* = 29) of patients. Patients with CR-POPF experienced significantly more insufficiencies of gastroenterostomies, delayed gastric emptying, and more extraluminal bleeding than patients without CR-POPF. Multivariate analysis revealed multiple postoperative predictive models, the best one including ASA, main pancreatic duct diameter, operation time, and serum lipase as well as leucocytes on day one. This model was able to predict CR-POPF with an accuracy of 90% and an AUC of 0.903. Two nomograms were created for easier use.

**Conclusion:**

Clinically relevant fistula can be predicted using simple laboratory and clinical parameters. Not serum amylase, but serum lipase is an independent predictor of CR-POPF. Our simple nomograms may help in the identification of patients for early postoperative interventions.

**Supplementary Information:**

The online version contains supplementary material available at 10.1007/s00423-021-02184-y.

## Introduction

Pancreatoduodenectomy (PD) represents the most common operation for either benign or malignant periampullary tumors. Since its introduction as Kausch-Whipple surgery in the early twentieth century, handling the pancreatic stump has been an issue. In fact, postoperative pancreatic fistula (POPF) remains the key complication with an incidence of up to 30% after pancreatic surgery, also in high volume centers [1, 2]. Clinically relevant POPF grade B/C (CR-POPF) are associated with post-pancreatectomy hemorrhage (PPH) and septic complications, which lead to prolonged hospital stay and high hospital costs [3, 4]. Late POPF detection delays necessary treatment options and poses a meaningful threat to perioperative survival [5]. In fact, up to 88% of perioperative deaths are caused by clinically relevant pancreatic fistula [3, 6]. Since preventive strategies, such as main duct drainage, the use of somatostatin analogues, or biological sealants are expensive and have failed to decrease POPF rates after PD, [7–10] the resection of the pancreatic remnant could be a safer option [6]. However, short-term risk must justify the long-term sequela with endocrine, exocrine pancreatic insufficiency as well as reduced quality of life [4]. However, the identification of patients with a CR-POPF versus a mere biochemical leak remains difficult. As the clinical management of CR-POPF implicates an early discovery, the understanding of perioperative risk factors and the underlying patho-mechanism is crucial.

Therefore, the aim of this study was to identify perioperative risk factors with an emphasis on postoperative laboratory parameters to facilitate prognosis of CR-POPF early in the postoperative period.

## Methods

This retrospective study was conducted between 2012 and 2017 at the University Medical Center Schleswig-Holstein, Campus Luebeck, Germany. Data were collected from a prospective institutional database. The study protocol was approved by the local ethical committee (trial number: 18-300A). From the 350 patients who underwent pancreatic resection, we included patients with non-pylorus-preserving pancreatic head resections (classical Whipple, PD) and patients with pylorus-preserving pancreaticoduodenectomy (PPPD) (*n*=182). An internal validation cohort from 2018 and 2019 was also analyzed. Of 228 patients with pancreatic resections in the 2-year time period, 90 patients underwent pancreatic head resection. We only included patients who had all baseline parameters and laboratory parameters included in the predictive model (POD1) available. Therefore, 48 patients without missing values were included in the validation cohort.

### Treatment and follow-up

All patients had preoperative abdomino-pelvic CT scan, and complete blood tests were performed 1 day prior to the operation. Pylorus-preserving PD was performed if it did not compromise resection margins. Pancreatogastrostomy (PG) was the preferred reconstruction technique for both open procedures and laparoscopic procedures. Sealants were not used. All of the patients were treated to our standardized postoperative protocol for pancreatic resections, including an overnight stay at the intensive care unit. Somatostatin analogues were used at the operating surgeon’s discretion. Outputs from operatively placed drains were recorded. Lipase, amylase, and bilirubin levels were measured daily or every other day until day five. Drains were removed on day three if volume was less than 200 ml and amylase levels were less than 300 U/l.

### Primary endpoint

POPF B/C was set as the primary endpoint of our study. POPF was classified using the ISGPF 2005 definition as an amylase activity in abdominal drain fluid three times the upper limit of the serum amylase on postoperative day three [7]. The upper limit for the serum amylase level in our institution is 100 U/l. did not use the 2017 classification because the data was prospectively collected before the new definition. To enable comparison with other studies, we separated the study collective into two subgroups: no POPF/ POPF A and POPF B/C. POPF B/C was defined as clinically relevant POPF (CR-POPF).

### Risk factors and secondary endpoints

A secondary endpoint was to develop a nomogram for easier clinical use. We evaluated clinical, patient-related variables such as BMI, ASA-classification (American Society of Anesthesiologists), need of care (measured according to WHO/ECOG Performance Status: 0–1—status 1 (self-sufficient), 2–3—status 2 (some care needed), 4—status 3 (full care needed)), comorbidities (diabetes mellitus, coronary heart disease, alcohol abuse), and preoperative medication (corticosteroids, immunosuppression). Preoperative laboratory values included C-reactive protein (CRP (mg/l)), white blood cell count (WBC in n/l), WBC-slope (meaning the highest difference between two WBC values in the first five postoperative days), hemoglobin (g/dl), creatinine (μmol/l), bilirubin (μmol/l), y-glutamyl transpeptidase (U/l), alkaline phosphatase (U/l), serum-amylase, and -lipase (U/l), and -slope (highest difference between two serum amylase or lipase values in the first five postoperative days). During surgery, surgical techniques (pancreatogastrostomy vs. pancreatojejunostomy), operation time (minutes), operation type (laparoscopic vs. open), pancreatic texture (soft vs. hard), and the main pancreatic duct diameter (MPD-diameter) were recorded. Normal MPD diameter was defined as less than 3 mm. The operating surgeon classified pancreatic texture intraoperatively as soft or hard. In the postoperative period serum urea, serum-, and drain- amylase (U/l), and lipase (U/l), WBC, and CRP were collected on postoperative days (POD) one to five. Postoperative complications (delayed gastric emptying, post-pancreatectomy hemorrhage, anastomotic insufficiencies) were classified according to the Clavien-Dindo Classification ^14^. In-hospital mortality (IHM) and post-pancreatectomy hemorrhage (PPH) were considered as secondary endpoints. PPH and delayed gastric emptying (DGE) were equally defined according to the International Study Group on Pancreatic Surgery ^16,17^. IHM was defined as death occurring during the overall hospital stay following pancreatoduodenectomy.

### Statistical methods

Statistical analysis was performed using the R software package glmnet ^14^ (R Version 3.4.4 (R Core Team 2017). Univariate analysis of 44 variables was conducted to identify perioperative risk factors for the development of POPF B/C. Statistically significant clinical and laboratory risk factors were used for risk stratification and model construction. For univariate analysis, median, minimum, and maximum were calculated. Statistical significance for all other analyses was defined as *p* ≤0.05. To identify the perioperative risk or predisposing factors of CR-POPF development, we performed a model-based general linear model with backward elimination leading to the construction of two predictive models. Multivariate binary logistic regression was calculated using the GLM (generalized linear model) method ^16^. All variables within the defined time frame with a *p*-value < 0.1 and less than 20% missing values were included. For the POD1 model ASA, need of care, MPD diameter, pancreatic texture, BMI, drain amylase on day one, operation time, serum lipase on day one, and white blood cell count on day one were included in the multivariate regression analyses. We entered ASA, need of care, MPD diameter, pancreatic texture, operation time, drain amylase on day one, serum lipase on day one, drain amylase slope, serum lipase slope, and white blood cell slope into the POD1-5 multivariate analysis. After backward elimination, the best performing model was based on the evaluation of the area under the receiver operating characteristic curve (AUC) and accuracy. Two nomograms were drawn from the POD1 and POD1-5 model in R®. These models allow for the calculation of the patient’s specific risk of developing a clinically relevant fistula. For example, when utilizing the POD1 nomogram, a chronic pancreatitis patient who had a main pancreatic duct < 3mm (normal), an OR time of 292 min, an ASA of III, a serum lipase of 102 U/l, and WBC of 8610/μl on POD1 had 121 cumulative points, resulting in a risk of about 10% to develop a CR-POPF. To note, this patient did not develop a pancreatic fistula after pancreatic head resection.

## Results

One hundred eighty-two patients underwent PD or pylorus-preserving pancreatoduodenectomy (PPPD) for benign or malignant tumors involving the head of the pancreas of the periampullary region at one tertiary center. The study cohort was composed of 82 women (45%) and 100 men (55%) with a median age of 66 years. Patient-related pre- and intraoperative clinical and laboratory characteristics are summarized in Table 1. The patients were relatively self-sufficient with only six patients (3%) in need of care. The median operation time was 362 min (range: 188–680 min). The surgery was done in open access in a little more than two-thirds of the time (69%) with PG (85%) as most common anastomotic reconstruction. One hundred and two (56%) patients received open PG reconstruction. Common pathologies were pancreatic ductal adenocarcinoma (PDAC) (38%), chronic pancreatitis (CP) (16%), and cystic neoplasms of the pancreas (12%).

**Table 1:** Univariate analysis of pre-/intraoperative patient characteristics and histology

	No POPF/ POPF A *n*=153 (84%)		POPF B/C *n* =29 (16%)		*p*-value
	Median/*N*	Range/%	Median/*N*	Range/%	
Preoperative patient characteristics					
Age, median (years)	66	(19–85)	65	(52–84)	0.94
Gender					
Male	81	53%	19	66%	0.296
Female	72	47%	10	34%	
Body mass index (kg/m^2^)	24.8	(16.33–42.02)	26.1	(15.47–37.11)	**0.043**
Need of care					
Partial/full	3	2%	3	10%	**0.08**
None	150	98%	26	90%	
ASA-classification					
I/II	87	57%	10	34%	**0.044**
III/IV/V	66	43%	19	66%	
Alcohol abuse	11	7%	0	0%	0.287
Weight loss	27	18%	4	14%	0.813
Diabetes mellitus	35	23%	10	35%	0.357
Coronary artery disease	17	11%	6	21%	0.263
Neoadjuvant radio-/chemotherapy	2	1%	1	3%	0.972
Preoperative corticosteroids	1	1%	1	3%	0.725
Preoperative imunosuppression	1	1%	0	0%	0.999
Preoperative laboratory variables					
C-reactive protein (mg/dl)	5.2	(0.1–287)	7.6	(0.6–98)	0.421
White blood cell count (n/l)	7185	(5–16,200	7745	(1140–16,490)	0.306
Hemoglobin (g/dl)	12.7	(6.9–19.3)	13.45	(9.3–29.9)	0.191
Creatinine (μmol/l)	0.81	(0.1–77.1)	0.84	(0.4–84.7)	0.809
Bilirubin (μmol/l)	2.52	(0.19–9174)	2.17	(0.46–2528)	0.484
γ-glutamyl transpeptidase (U/l)	88.5	(10–3959)	156	(9.19–1406)	0.472
Alkaline phosphatase (U/l)	163	(1–1814)	252	(45–697)	0.972
Amylase (U/l)	59	(1.4–443)	103	(18–464)	**0.041**
Lipase (U/l)	51	(1–1259)	47.5	(4–999)	0.701
Intraoperative characteristics					
Operation time (minutes)	359	(188–680)	387	(262–578)	**0.032**
Surgical procedure					0.853
Laparoscopic/assisted	48	31%	8	28%	
Open	105	69%	21	72%	
Pancreatic anastomosis					0.683
Closure	1	1%	0	0%	
PG	128	84%	26	90%	
PJ	24	16%	3	10%	
Portal vein resection	26	17%	3	10%	0.535
Intestinal resection	1	1%	0	0%	0.999
Main pancreatic duct diameter					**< 0.001**
Normal	75	49%	28	97%	
Dilated	78	51%	1	3%	
Pancreatic texture					**< 0.001**
Hard	83	54%	2	7%	
Soft	70	46%	27	93%	
Intraoperative red blood cell transfusion	0	(0–8)	0	(0–6)	0.157
Histological diagnosis					**0.093**
Ampullary adenocarcinoma	10	7%	2	7%	
Ductal adenocarcinoma	62	41%	8	28%	
Distal bile duct Adenocarcinoma	8%	5%	2	7%	
Duodenal adenocarcinoma	4	3%	1	3%	
Neuroendocrine tumor	9	6%	3	10%	
Cystic pancreatic neoplasm	17	11%	4	14%	
Chronic pancreatitis	29	19%	1	3%	
Pancreatic cyst	1	1%	0	0%	
Others	13	8%	8	28%	

*ASA* American Society of Anesthesiologists, *PG* pancreatogastrostomy, *PJ* pancreatojejunostomy

Significant values are shown in bold

The overall rate of POPF in our study was 36% (66 patients) and clinically relevant POPF (CR-POPF ((ISGPF 2005 grade B and C)) occurred in 16% (29 patients) of patients. To identify risk factors for POPF in our cohort, patients who presented CR-POPF (*n*=29) and those who did not (*n*=153) were compared (Tables 1, 2, and 3). Factors positively correlating with CR-POPF in the preoperative period included higher BMI (26.1 [range: 15.5–37.1] vs. 24.8 [range: 16.3–42.0]; *p*=0.043), higher ASA classification (grade III/IV) (66% [*n*=19] vs. 43% [*n*=66]; *p*=0.044), any need of care (partial and full) (10% [*n*=3] vs. 2% [*n*=3]; *p*=0.08), and higher (yet almost within the normal range of 100 U/l defined by our institutional laboratory) preoperative serum-amylase (103 U/l [range: 18–464] vs. 59 U/l [range: 1.4–443]; *p*=0.041). Pertaining the intraoperative risk factors, a normal MPD-diameter (<3 mm) (97% [*n*=28] vs. 49% [*n*=75]; *p*<0.001), soft pancreatic texture (93% [*n*=27] vs. 46% [*n*=70]; *p*<0.001), less fibrotic histological diagnosis (PDAC and chronic pancreatitis) (31% [*n*=9] vs. 59% [*n*=91]; *p*=0.009), and longer operation time (387 min. [range: 262–578] vs. 359 min. [188–680]; *p*=0.032) were associated with higher rates of CR-POPF. We observed no difference in the mode of reconstruction (pancreatogastrostomy (90% [*n*=26] vs. 84% [*n*=128]; *p* = 0.683) between patients with and without CR-POPF. This was also true, if we subdivided our patient cohort in laparoscopic/assisted (lap/ass) access (PG 86% [*n*=45] in the no POPF/A group vs. 75% PG [*n*=3] in the POPF B/C group; *p* = 0.525) and in open access (PG 81% [*n*=83] vs. PG 91% [*n*=21]; p = 0.464), respectively to avoid comparative bias.

**Table 2:** Univariate analysis of postoperative patient characteristics

	No POPF/ POPF A *n*=153 (84%)		POPF B/C *n* =29 (16%)		*p*-value
	Median/*N*	IQR/%	Median/*N*	IQR/%	
CDC classification					**< 0.001**
0	37	24%	0	0%	
1	10	7%	0	0%	
2	55	36%	2	7%	
3a	17	11%	10	34%	
3b	17	11%	5	17%	
4a	14	9%	6	21%	
4b	2	1%	1	3%	
5	1	1%	5	17%	
Intraabdominal abscess	7	5%	21	72%	**< 0.001**
Bile duct anastomosis leak	9	6%	5	17%	0.085
Gastroenterostomy leak	4	3%	4	14%	**0.028**
Burst abdomen	6	4%	4	14%	0.09
SSI with wound reopening	21	14%	12	41%	**0.001**
DGE					**< 0.001**
None/ DGE A	136	89%	15	52%	
DGE B/C	17	11%	14	48%	
PPH					0.086
None/ PPH A/B	141	92%	23	79%	
PPH C	12	8%	6	21%	
PPH intraluminal	22	14%	2	7%	0.428
PPH extraluminal	5	3%	6	21%	**0.001**
Unplanned ventilation	10	7%	9	31%	**< 0.001**
Postoperative hemodialysis	0	0%	5	17%	**< 0.001**
Pneumonia	26	17%	14	48%	**< 0.001**
Discharge circumstance					**<0.001**
Home	125	82%	20	69%	
Hospice/death	1	1%	6	21%	
Hospital	13	8%	0	0%	
Rehabilitation	14	9%	3	10%	
Reoperation	37	24%	18	62%	**< 0.001**
Histology risk group					**0.009**
Yes	62	41%	20	69%	
No	91	59%	9	31%	
Readmission	19	12%	7	24%	0.172
ICU stay days	1	0–51	4	1–79	**< 0.001**
IHM	1	1%	5	17%	**< 0.001**

*CDC* Clavien-Dindo classification of complications, *SSI* surgical site infection, DGE delayed gastric emptying, PPH post-pancreatectomy hemorrhage, *ICU* intensive care unit, IHM in-hospital mortality

Significant values are shown in bold

**Table 3:** Laboratory values on postoperative day one, two, three, four, and five

	No POPF/ POPF A *n*=153 (84%)		POPF B/C *n* =29 (16%)		*p*-value	Missing values (*n*)
	Median/*N*	IQR/%	Median/*N*	IQR/%		
Serum amylase (u/l) day 1	35	(5–7500)	264	(233–579)	0.086	173
Drain amylase (u/l) day 1	136	(3–73,614)	4017.5	(132–28,505)	< 0.001	11
Serum lipase (u/l) day 1	60	(3–1537)	450.5	(48–1614)	< 0.001	5
Drain lipase (u/l) day 1	451	(4–21,900)	9643	(506–31,662)	< 0.001	65
CRP (mg/l) day 1	79.8	(6–306)	96.2	(69.6–258)	0.059	112
WBC (n/l) day 1	12,080	(5730–32,240)	14,780	(7310–27,080)	0.036	2
Urea (mmol/l) day 1	5.095	(2.38–14.48)	4.47	(3.43 – 7.9)	0.712	143
Serum amylase day 2	25.5	(5–300)	264	(124–5803	0.012	149
Serum amylase day 3	31.5	(3–280)	57.5	(51–64)	0.441	156
Serum amylase day 4	20	(5–331)	54.5	(15–189)	0.036	152
Serum amylase day 5	22.5	(9–87)	37	(8–179)	0.291	165
Drain amylase day 2	146	(3–24,162)	4370	(248–75,000)	<0.001	31
Drain amylase day 3	47	(3–5573)	1108	(37–4941)	<0.001	53
Drain amylase day 4	31	(3–3900)	342.5	(9–4777)	<0.001	78
Drain amylase day 5	21	(3–3898)	107	(3–1951)	0.022	89
Serum lipase day 2	25	(2–696)	161	(8–2009)	<0.001	65
Serum lipase day 3	14	(4–221)	69.9	(6–597)	<0.001	103
Serum lipase day 4	9.5	(4–101)	21	(6–559)	<0.001	118
Serum lipase day 5	13	(4–137)	22	(4–1051)	0.383	125
Drain lipase day 2	631	(3–30,000)	15,150	(411–63,728)	<0.001	83
Drain lipase day 3	107	(4–30,000)	5017	(538–20,200)	<0.001	123
Drain lipase day 4	35.5	(3–9600)	1500	(41–5682)	<0.001	138
Drain lipase day 5	64	(4–11,800)	742	(159–9177)	0.006	125
CRP day 2	165.5	(47.1–320)	186.2	(73.9–303)	0.680	78
CRP day 3	133	(34.8–428)	217.5	(62.9–334)	0.029	84
CRP day 4	123	(19.5–350)	196.8	(43.6–427)	0.001	92
CRP day 5	76	(15.2–306)	170.5	(58.2–336.6)	0.001	105
WBC day 2	11,880	(4810–28,280)	15,055	(8320–35,220)	0.009	25
WBC day 3	10,640	(4540–23,260)	13,700	(4380–29,240)	0.004	45
WBC day 4	9380	(4920–22,790)	11,940	(5750–23,190)	<0.001	49
WBC day 5	9595	(4230–20,060)	14,795	(7550–27,539)	<0.001	72
Urea day 2	6.45	(2.44–2.5)	8.88	(5.2–12.3)	0.102	132
Urea day 3	5.925	(3.08–8.7)	6.96	(4.69–12.8)	0.147	132
Urea day 4	5.425	(2.7–35.56)	6.17	(3.93–7.91)	0.642	158
Urea day 5	5.23	(2.9–18.38)	7.9	(4.12–15.8)	0.644	169

*WBC* white blood cell count, *CRP* C-reactive protein

In the postoperative period, CR-POPF was associated with general surgical complications such as surgical site infection with need of wound reopening (41% [*n*=12] vs. 14% [*n*=21]; *p*= 0.001) or pneumonia (48% [*n*=14] vs. 17%. [*n*=26]; *p*<0.001) (Table 2). Furthermore, we found that specific procedure-complications such as extraluminal post-pancreatectomy hemorrhage (PPH) (*p*<0.001), delayed gastric emptying (DGE) B/C (*p*<0.001), intraabdominal abscesses (*p*<0.001), and leaks of the gastroenterostomy (*p*=0.028) were significantly associated with CR-POPF. Consequently, patients who developed CR-POPF had higher grades in the CDC-classification (Clavien-Dindo classification of surgical complications) (*p*<0.001), a longer intensive care unit (ICU) stay (4 days [range: 1–79] vs. 1 day [range: 0–51]; *p*<0.001), and were subjected more often to intensive care procedures such as postoperative hemodialysis (17% [*n*=5] vs. 0% [*n*=0]; <0.001) or unplanned ventilation (31% [*n*=9] vs. 7% [*n*=10]; *p*<0.001). The overall in-hospital mortality (IHM) during the hospital stay was 3% (*n*=6). IHM in patients with CR-POPF was significantly higher than in patients with no POPF or POPF A (17% [*n*=5] vs. 1% [*n*=1]; *p*<0.001). We further evaluated laboratory parameters on postoperative day one (POD1) (Table 3). Median drain amylase (4018 U/l [range:132–28,505] vs. 136 U/l [range:3–73,614]; *p*<0.001) and lipase (9643 U/l [range: 506–31,662] vs. 451 U/l [range: 4–21,900]; *p*<0.001) on POD1 were significantly higher in patients with POPF B/C. Correlation between laboratory parameters and CR-POPF was found for serum lipase (451 U/l [range: 48–1614] vs. 60 U/l [range: 3–1537]; *p*<0.001), but not serum amylase (264 U/l [range: 233–579] vs. 35 U/l [range: 5–7500]; *p*=0.086), and WBC count (14,780 n/l [range: 7310–27,080] vs. 12,080 n/l [range: 5730–32,240]; *p*=0.036) on POD1. Serum urea did not show a significant correlation with the occurrence of CR-POPF (*p*=0.712). However, missing data was high for serum urea and patients in which serum urea was measured and those where it was not measured differed in many pancreatic fistula–related parameters. Therefore, we cannot exclude serum urea as a POPF prognostic value based on our data.

### Multivariate analysis for prediction model building

**Fig. 1:**
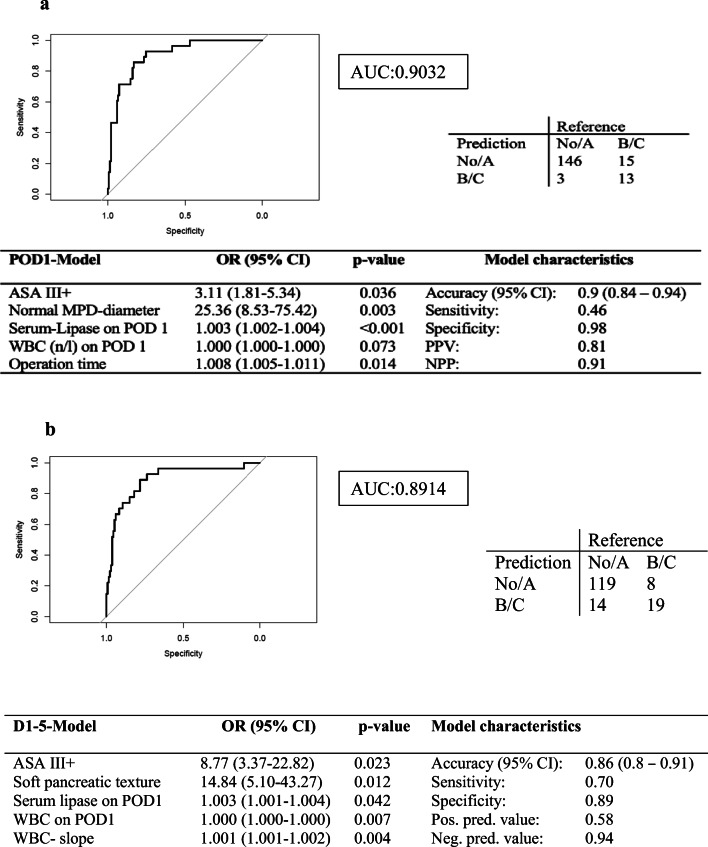
Multivariate generalized linear models for the prediction of clinically relevant fistula**. a** POD1 model. Only variables that can be assessed before or up until postoperative day one are included. **b** POD1-5 model. Only variables that can be assessed before or up until postoperative day five are included. WBC, white blood cell count/μl; OP, operation; ASA, American Society of Anesthesiology classification; MPD, main pancreatic duct; d1, postop day1; Serum lipase.d1 on postoperative day one in U/l

The early postoperative model (POD1 model) identified ASA classification >III (OR: 3.11), MPD-diameter (OR: 25.36), operation time (OR: 1.008), serum-lipase (OR: 1.003), and WBC count (OR: 1.000) on POD1 (Fig. 1a) as independent risk factors for POPF prediction. The POD1 model had an accuracy of 89.8% and an AUC of 0.903 (95% CI: 0.84–0.94), respectively. Due to moderate sensitivity (46.43%) and high specificity (97.99), 13 of 28 POPF B/C were captured by the model, but only three patients were predicted to have a CR-POPF but did not develop one. Correspondingly, the negative predictive value of this model was high, with a value of 91%. We also built a model (POD1-5 model) that included parameters from the first five postoperative days including ASAIII and above (OR 8.77), soft pancreatic texture (OR 14.84), serum lipase on POD1 (OR 1.003), WBC on POD1 (OR 1.00), and WBC-slope (OR 1.00) after backward elimination. Accuracy was 86%, and sensitivity was 70% with a specificity of 89%.

**Fig. 2:**
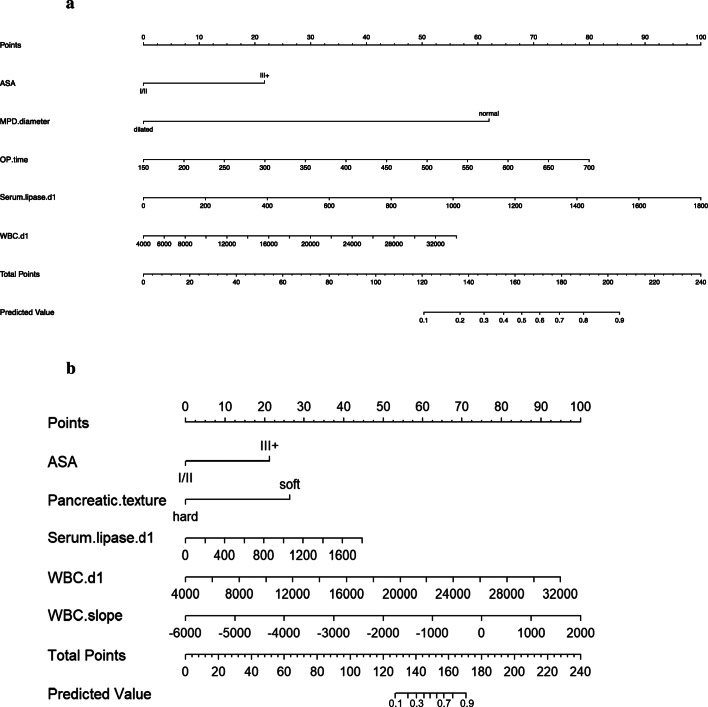
**a** POD 1 nomogram: a tool to calculate the risk of CR-POPF on postoperative day one. (Predictive value*100= %risk). The reference line is used for reading scoring points (default range 0–100) for each variable. Once the reader manually totals the points, the predicted values can be read at the bottom (applies to both nomograms). **b** POD1-5 nomogram. WBC, white blood cell count/μl; OP, operation; ASA, American Society of Anaesthesiology classification; MPD, main pancreatic duct; d1, postoperative day 1; serum lipase.d1 on postoperative day one in U/l

**Fig. 3:**
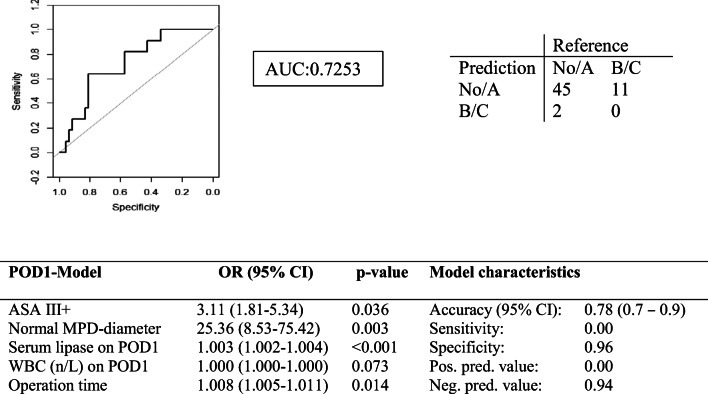
Prediction model POD1 in the validation cohort. WBC, white blood cell count/μl; ASA, American Society of Anesthesiology classification; MPD, main pancreatic duct;  POD, postoperative day1; serum lipase on postoperative day one in U/l

In order to possibly utilize our data in the clinic, we developed two nomograms from the POD1 model (Fig. 2a) and POD1-5 model (Fig. 2b). We also validated the early postoperative model (POD1 model) internally by analyzing a separate patient cohort from 2018 and 2019. Baseline characteristics of 48 patients are shown in supplementary Table 1. The POD1 model had an AUC of 0.725 and an accuracy of 0.779 (Fig. 3) in the validation cohort.

## Discussion

Despite advances in surgical techniques, newly identified pancreatic fistula risk scores and the use of somatostatin analogues, CR-POPF remains the most relevant clinical hazard in pancreatic head resections [11]. Of course, based on the definition of the ISGPS, on postoperative day three, the presence of a pancreatic fistula is evident; however, no correlation with the severity can be concluded from the definition or the number value of drain and serum amylase. Thus, the reliable identification of high-risk symptomatic patients and a better understanding of the pathophysiology may not only improve postoperative outcomes in terms of morbidity but are also of great interest in the adjustment of the early clinical management.

Risk factors for pancreatic fistula per se have been widely described in the literature. However, most interest should be directed toward clinically relevant symptomatic pancreatic fistula as highlighted in the new 2016 classification of the ISGPS [12] and because these are the main causes of perioperative morbidity. Our data is in line with the validated clinical risk factors that include high BMI, operative time, soft pancreatic texture, small pancreatic duct diameter, and histopathological diagnosis other than pancreatic fibrosis or pancreatic adenocarcinoma. In addition to the mentioned findings, our data identified several new factors including need of care and ASA classification (>III) as independent preoperative risk factors for CR-POPF. This finding is interesting, as one would assume these patients to be older (although age did not independently correlate with CR-POPF development), more often smokers, and possibly diabetic, and therefore incorporate a more fibrotic and atrophied gland. Arguably, this protective factor is overruled by something else. The immunologic response might be impaired as patients needing special care at home or in a nursing home are more prone to infections [13–15]. Conversely, patients receiving neoadjuvant therapy, also suffering from an impaired immunologic status, display significantly less clinically relevant pancreatic fistula [16]. However, if they develop POPF anyway, their survival is largely reduced, suggesting a lacking rescue mechanism of this complication, possibly also responsible for our observation.

Elevated serum- and drain-amylase-level in the early postoperative period have been identified as parameters indicating the occurrence of pancreatic fistula in several trials [11, 12], whereas lipase has only been rarely mentioned in this context. Completing a large retrospective trial of 2007 [17], Bassi et al. concluded in a prospective trial that serum-amylase on POD1 showed a significant association with CR-POPF (*p*=0.001). Another trial found a significant association between elevated serum-amylase on the night of surgery and elevated CRP-levels on POD2 with CR-POPF. As for the determination of a valid cut-off for POD1 drain-amylase, there still exist conflicting results.

In our univariate analysis, only drain-amylase not serum amylase was significantly correlated with CR-POPF. However, neither serum- nor drain-amylase was selected in multivariate analysis as a predictive factor. This can be due to the fact that amylase is only an indicator of a non-specific reaction to intraoperative pancreatic trauma inducing the release of pancreatic enzymes. In fact, serum-amylase is not a POPF specific laboratory value, as it can also be elevated in other pancreas-related diseases. In addition, our data for serum amylase had many missing values, but patient cohorts with measurements of serum amylase and those without did not differ systematically confirming the validity of the data (data shown in supplementary files).

On the contrary, patients with serum-lipase levels < 44.5 U/l on POD1 did not develop POPF in the majority of the cases, suggesting a highly predictive role for serum lipase in the postoperative period. Supporting these findings, we found that median levels of serum-lipase were significantly higher in patients with CR-POPF than in patients who had POPF A or no POPF. While technical aspects of pancreatic surgery, especially the choice of the anastomotic techniques have long been assumed to play a key role in the occurrence of pancreatic fistula, no specific surgical technique could prove to be superior to another. However, recent findings suggest an inflammatory mechanism in the sense of postoperative pancreatitis as main reason for CR-POPF [18], shedding light on the pathophysiology of this deadly complication. One part of the hypothesis is that transient hypoperfusion of the pancreatic remnant due to pancreatic infarction caused by interrupted blood flow during surgery plays an important role in the pathway leading to acute postoperative pancreatitis. Serum lipase elevation could therefore just be an indicator of pancreatitis of the pancreatic remnant, pointing towards pancreatitis (mainly post-ERCP pancreatitis) directed therapy for pancreatic fistula. For example, oxygen-derived free radicals contribute to the pathogenesis of acute pancreatitis by inducing capillary-endothelial injury, leading to increased capillary permeability [19]. Drugs that prevent the generation of/and/or inactivate free radicals include allopurinol and n-acetylcysteine, respectively. Unfortunately, in post-ERCP pancreatitis, these two medications did not show any clear benefit in randomized controlled trials [20–23]. The usage of corticosteroids as inhibitors of the inflammatory response has shown mixed results [24, 25]. Surprisingly, a single dose of hydrocortisone was able to reduce post-ERCP pancreatitis from 12 to 2% in the treatment group [26]. Similarly, a single dose of dexamethasone (as postoperative nausea and vomiting (PONV) prophylaxis) intraoperatively decreased the rates of infectious complications as well as prolonged long-term survival after pancreatoduodenectomy for PDAC [27]. Correspondingly, the risk-adjusted administration of hydrocortisone resulted in fewer pancreatic fistula (11 vs. 27%) and overall complications after pancreatoduodenectomy [28] and distal pancreatectomy [29] in two Finnish randomized controlled trials, suggesting the safe use of corticosteroids for patients at high risk for pancreatic fistula, but this needs to be investigated by large randomized controlled trials. The attenuation of the inflammatory response is the target in NSAID therapy. Diclofenac, administered by rectal route, was able to reduce the incidence of post-ERCP pancreatitis by almost 50% in two randomized controlled trials [30, 31]; however, diclofenac itself can rarely cause pancreatitis, warranting caution for the use in the early postoperative period [32]. Another treatment option of post-ERCP pancreatitis possibly interesting for the treatment of pancreatic fistula is to interrupt the protease activity by protease inhibition via heparin, ulinastatin, or gabexate maleate [33–36]. In post-ERCP pancreatitis, results are conflicting; however, there have not been any studies for pancreatic fistula treatment. All of these treatments need early initiation and could be allocated based on our risk model (POD1 or POD1-5).

Considering postoperative complications, DGE, intraabdominal abscess, bile duct anastomosis leak or insufficiencies of the gastroenterostomy, and PPH have been frequently associated with CR-POPF. The correlation with CR-POPF has been examined in various trials and it has been shown that severe PPH showed significant association with CR-POPF. In our study, we were able to confirm these findings as PPH C occurred in 18 patients (10%) of the overall patient collective while 21% of the patients with PPH C had also POPF B/C. In order to dissect this finding even further, we investigated the cause of bleeding and defined two categories: extraluminal bleeding and intraluminal bleeding. Intraluminal bleeding appears in general within the first postoperative day and is caused by technical failure or insufficient hemostasis requiring relaparotomy; it is also more common after pancreatogastrostomy versus pancreatojejunostomy due to the freely floating pancreatic stump within the stomach [37]. In contrast, extraluminal bleeding occurs in the period after 24h and is mostly caused by a rupture of pancreatic blood vessels originating from the development of pancreatic fistula or ulceration. While intraluminal bleeding showed no association to the occurrence of CR-POPF in our study, extraluminal bleeding was strongly associated with CR-POPF (*p* = 0.001). It might be speculated that extraluminal bleeding occurs in the context of the development of postoperative pancreatitis and leaking pancreatic enzymes leading to vessel erosion more than 24h after surgery, suggesting a prophylactic stent placement in high-risk patients as treatment option.

Existing prediction models like the fistula risk score [11] and the alternative fistula risk score [38] found the fistula risk score and drain-amylase of POD1 as equally predictive tools for POPF prediction. Comparing our results to other reported predictive models, we included clinical and laboratory values and were able to build a highly predictive model on POD1 with a high AUC, moderate sensitivity, and high specificity. A model with high specificity and low sensitivity, such as our POD1 model, can be useful to rule out CR-POPF, making it a potential tool for patient allocation for ERAS at low risk for CR-POPF. ERAS programs have been implemented for many different procedures; however, in pancreatic centers, they are slow to find acceptance [39, 40]. Nevertheless, hospital duration and postoperative complications Clavien-Dindo stage I–II can be reduced by ERAS [41]. Accordingly, low-risk patients may benefit from the ERAS protocol [42].

Furthermore, our predictive models and nomograms allow for the opportunity to calculate the individual risk for the development of CR-POPF. It is unclear what cutoff should be used to consider change of treatment strategy, possibly also depending on the individual’s age. A risk above 90% (from the nomogram) at a positive predictive value of 81% translates into a cumulative risk of ca. 73% for the development of CR-POPF, which we consider the minimum risk to alter treatment decision in the direction of drastic “remnant pancreatectomies.” The calculation on POD1 enables the responsible physician to make a treatment decision in the early postoperative period before the patient becomes septic. Early remnant pancreatectomy can lead to less morbidity and mortality caused by pancreatic fistula [5, 6]. However, we need to consider the time point at which the decision for interventional drainage or relaparotomy is made. When decision-making is postponed to the time point, when clinically relevant or “severe” pancreatic fistula is already present, primary relaparotomy is associated with higher morbidity and mortality and interventional drainage is preferred [43].

Limitations of this study include the retrospective nature of the analysis and the usage of the old ISGPS classification for pancreatic fistula. Missing values might skew the data. In addition, the validation cohort is small and should be evaluated with caution. We did not include variables with more than 20% missing values into our multivariate model, which excluded serum amylase, serum urea, and serum CRP on postoperative day one already before we even started the calculation. However, we performed secondary analysis of our data to test if the patient cohorts differed between those in which laboratory parameters were missing and those where it was not missing. We found no systemic differences between the two cohorts (shown in Supp. Tables 2 and 3 exemplary for serum amylase on postoperative day one). Based on this data, we can at least assume that our data is valid. However, to prove that serum amylase, serum urea, and serum CRP on postoperative day one do not present valid POPF predictors, larger studies should be performed.

## Conclusion

Our two early postoperative nomograms are able to calculate the risk for the development of clinically relevant postoperative pancreatic fistula, and may help in the identification of patients for early postoperative interventions. In addition, serum lipase on postoperative day one is independently associated with clinically relevant POPF.

## Supplementary Information

ESM 1 (DOCX 18 kb)

ESM 2 (DOCX 35 kb)
