# Supplementary material for: A simple nomogram for early postoperative risk prediction of clinically relevant pancreatic fistula after pancreatoduodenectomy
Source: Langenbecks Arch Surg. 2021 May 19;406(7):2343–55. doi: 10.1007/s00423-021-02184-y (PMC8578094; doi:10.1007/s00423-021-02184-y)
Supplement: Supplementary file 1 — (DOCX 18 kb) [file 423_2021_2184_MOESM1_ESM.docx]

Supplementary Table 1: Validation cohort (48 patients with PD or PPPD)

Data is presented as n (%) or median (IQR)- There are no missing values.

|  | Total  (n=48) | No POPF/POPF A  (n=39) | POPF B/C  (n=9) | p-value |
| --- | --- | --- | --- | --- |
| Gender |  |  |  | 0.999 |
| Female | 17 (35%) | 14 (36%) | 3 (33%) |  |
| Male | 31 (65%) | 25 (64%) | 6 (67%) |  |
| Body mass index (kg/m^2^) | 24.2 (5.6) | 23.4 (6.2) | 25.8 (3.4 | 0.335 |
| ASA-classification |  |  |  | 0.859 |
| I/II | 9 (19%) | 8 (21%) | 1 (11%) |  |
| III/IV/V | 39 (81%) | 31 (79%) | 8 (89%) |  |
| Need of care |  |  |  | 0.999 |
| None | 47 (98%) | 38 (97%) | 9 (100%) |  |
| Partial/full | 1 (2%) | 1 (3%) | 0 (0%) |  |
| Surgical procedure |  |  |  | 0.595 |
| Laparoscopic/-assisted | 31 (65%) | 24 (62%) | 7 (78%) |  |
| Open | 17 (35%) | 15 (38%) | 2 (22%) |  |
| Pancreatic texture |  |  |  | **0.012** |
| Hard | 26 (54%) | 25 (64%) | 1 (11%) |  |
| Soft | 22 (46%) | 14 (36%) | 8 (89%) |  |
| Main pancreatic duct diameter |  |  |  | 0.244 |
| Normal | 21 (44%) | 15 (38%) | 6 (67%) |  |
| Dilated | 27 (56%) | 24 (62%) | 3 (33%) |  |
| Operation time (minutes) | 372 (116) | 367 (109) | 399 (75) | 0.165 |
| Drain amylase (U/L) Day 1 | 135 (2253) | 100 (827) | 2205 (5801) | 0.055 |
| Serum lipase (U/L) Day 1 | 56 (281) | 49 (251) | 150 (326) | 0.355 |
| WBC (n/L) Day 1 | 10605 (4255) | 10560 (452) | 10730 (147) | 0.588 |
| Histological diagnosis |  |  |  | 0.637 |
| Pancreatic adenocarcinoma | 20 (42%) | 15 (38%) | 5 (56%) |  |
| Ampullary adenocarcinoma | 4 (8%) | 4 (10%) | 0 (0%) |  |
| Distal bile duct adenocarcinoma | 5 (10%) | 3 (8%) | 2 (22%) |  |
| Duodenal adenocarcinoma | 3 (6%) | 3 (8%) | 0 (0%) |  |
| Neuroendocrine tumor | 2 (4%) | 2 (5%) | 0 (0%) |  |
| Chronic pancreatitis | 8 (17%) | 7 (18%) | 1 (11%) |  |
| Others | 6 (12%) | 5 (13%) | 1 (11%) |  |
| POPF |  |  |  | **<0.001** |
| No | 34 (71%) | 34 (87%) | 0 (0%) |  |
| A | 5 (10%) | 5 (13%) | 0 (0%) |  |
| B | 3 (6%) | 0 (0%) | 3 (33%) |  |
| C | 6 (12%) | 0 (0%) | 6 (67%) |  |
|  |  |  |  |  |
|  |  |  |  |  |
|  |  |  |  |  |
|  |  |  |  |  |
|  |  |  |  |  |
| CDC-Classification |  |  |  | **0.018** |
| 0 | 10 (21%) | 10 (26%) | 0 (0%) |  |
| 1 | 3 (6%) | 3 (8%) | 0 (0%) |  |
| 2 | 12 (25%) | 12 (31%) | 0 (0%) |  |
| 3a | 3 (6%) | 1 (3%) | 2 (22%) |  |
| 3b | 7 (15%) | 6 (15%) | 1 (11%) |  |
| 4a | 2 (4%) | 1 (3%) | 1 (11%) |  |
| 4b | 7 (15%) | 4 (10%) | 3 (33%) |  |
| 5 | 4 (8%) | 2 (5%) | 2 (22%) |  |

CDC Clavien-Dindo-classification of complications; POPF postoperative pancreatic fistula; ASA American Society of Anaesthesiologists; WBC white blood cell count
