# Supplementary material for: A simple nomogram for early postoperative risk prediction of clinically relevant pancreatic fistula after pancreatoduodenectomy
Source: Langenbecks Arch Surg. 2021 May 19;406(7):2343–55. doi: 10.1007/s00423-021-02184-y (PMC8578094; doi:10.1007/s00423-021-02184-y)
Supplement: Supplementary file 2 — (DOCX 35 kb) [file 423_2021_2184_MOESM2_ESM.docx]

| **Supplementary Table 2: Baseline demographics between patients with serum amylase or not** | **Serum amylase POD1 not**  **measured**  n=172 (96%) | | | | | **Serum amylase POD1 measured**  n =10 (4%) | | | |  | | |
| --- | --- | --- | --- | --- | --- | --- | --- | --- | --- | --- | --- | --- |
|  | **Median/N Range/%** | | | | | **Median/N Range/%** | | | | **P-Value** | | |
| **Preoperative patient characteristics** | |  | |  | |  | |  | | | |  |
| Age, median (years) | | | 66 | | (19 - 85) | | 67 | | (41 - 78) | | 0.764 | |
| Gender | | |  | |  | |  | |  | |  | |
| male | | | 95 | | 45 % | | 5 | | 50 % | | 0.746 | |
| female | | | 77 | | 55 % | | 5 | | 50 % | |  | |
| Body mass index (kg/m^2^) | | | 24.9 | | (115.47 - 42.02) | | 27 | | (22.66 – 32.62) | | 0.108 | |
| Need of care | | |  | |  | |  | |  | |  | |
| partial/full  none | | | 5  167 | | 3 %  97 % | | 1  9 | | 10 %  90 % | | 0.222 | |
| ASA-classification | | |  | |  | |  | |  | |  | |
| I/II | | | 92 | | 53 % | | 5 | | 50 % | | 0.830 | |
| III/IV/V | | | 80 | | 47 % | | 5 | | 50 % | |  | |
| Alcohol abuse | | | 11 | | 6 % | | 0 | | 0 % | | 0.287 | |
| Weight loss | | | 31 | | 18 % | | 0 | | 0 % | | 0.171 | |
| Diabetes mellitus | | | 42 | | 24 % | | 3 | | 30 % | | 0.357 | |
| Coronary artery disease | | | 19 | | 11 % | | 4 | | 40 % | | **0.025** | |
| Neoadjuvant radio-/chemotherapy | | | 3 | | 2 % | | 0 | | 0 % | | 0.674 | |
| Preoperative corticosteroids | | | 1 | | 1 % | | 1 | | 10 % | | 0.107 | |
| Preoperative imunosuppression  **Preoperative laboratory variables** | | | 1 | | 1 % | | 0 | | 0 % | | 0.999 | |
| C-reactive protein (mg/dL) | | | 5.5 | | (0.1 - 287) | | 3.2 | | (1.7- 126) | | 0.739 | |
| White blood cell count (n/L) | | | 7105 | | (5 - 16490 | | 9055 | | (6980 – 15890) | | **0.006** | |
| Hemoglobin (g/dL) | | | 12.8 | | (6.9 - 29.9) | | 13.2 | | (11.2 – 15.4) | | 0.364 | |
| Creatinine (μmol/L) | | | 0.81 | | (0.1 – 84.7) | | 0.80 | | (0.5 – 1.2) | | 0.871 | |
| Bilirubin (μmol/L) | | | 2.52 | | (0.19 – 9.17) | | 2.17 | | (0.32 – 15.04) | | 0.894 | |
| γ-glutamyl transpeptidase (U/L) | | | 91.5 | | (10 - 3959) | | 84 | | (9.19 - 2327) | | 0.971 | |
| Alkaline phosphatase (U/L) | | | 170 | | (1 - 1814) | | 152 | | (57 - 384) | | 0.897 | |
| Amylase (U/L) | | | 59 | | (1.4 - 464) | | 94 | | (13 - 224) | | 0.180 | |
| Lipase (U/L) | | | 51 | | (1 - 1259) | | 40 | | (4 - 999) | | 0.649 | |
| **Intraoperative characteristics** | | |  | |  | |  | |  | |  | |
| Operation time (minutes) | | | 362 | | (188 - 680) | | 357 | | (235 - 510) | | 0.855 | |
| Surgical procedure | | |  | |  | |  | |  | | 0.957 | |
| laparoscopic/-assisted | | | 53 | | 31 % | | 3 | | 30 % | |  | |
| open | | | 119 | | 69 % | | 7 | | 70 % | |  | |
| Pancreatic anastomosis | | |  | |  | |  | |  | | 0.878 | |
| Closure | | | 1 | | 1% | | 0 | | 0% | |  | |
| PG | | | 145 | | 84 % | | 9 | | 90 % | |  | |
| PJ | | | 26 | | 16% | | 1 | | 10 % | |  | |
| Portal vein resection | | | 27 | | 17 % | | 3 | | 10 % | | 0.718 | |
| Intestinal resection | | | 1 | | 1 % | | 2 | | 20 % | | 0.809 | |
| Main pancreatic duct diameter | | |  | |  | |  | |  | | 0.665 | |
| normal | | | 98 | | 43 % | | 5 | | 50 % | |  | |
| dilated | | | 74 | | 43 % | | 5 | | 50 % | |  | |
| Pancreatic texture | | |  | |  | |  | |  | | 0.830 | |
| hard | | | 80 | | 46 % | | 5 | | 50 % | |  | |
| soft | | | 92 | | 54 % | | 5 | | 50 % | |  | |
| Intraoperative red blood cell transfusion | | | 0 | | (0 - 8) | | 0 | | (0 - 4) | | 0.952 | |
| Histological diagnosis | | |  | |  | |  | |  | |  | |
| Ampullary adenocarcinoma | | | 10 | | 7 % | | 0 | | 0 % | |  | |
| Ductal adenocarcinoma | | | 65 | | 38 % | | 5 | | 50 % | |  | |
| Distal bile duct Adenocarcinoma | | | 9 | | 5 % | | 1 | | 10 % | |  | |
| Duodenal adenocarcinoma | | | 5 | | 3 % | | 0 | | 30% | |  | |
| Neuroendocrine tumor | | | 12 | | 7 % | | 0 | | 0 % | |  | |
| Cystic pancreatic neoplasm | | | 20 | | 12 % | | 0 | | 0 % | |  | |
| Chronic pancreatitis | | | 27 | | 16 % | | 3 | | 30 % | |  | |
| Pancreatic cyst | | | 1 | | 1 % | | 0 | | 0 % | |  | |
| Others | | | 21 | | 12 % | | 0 | | 0 % | |  | |

| **Supplementary Table 3. Univariate analysis of postoperative patient characteristics- serum amylase yes or no** | | | | | |  |
| --- | --- | --- | --- | --- | --- | --- |
|  | **Serum amylase POD1**  **not measured**  n=172 (96%) | | | **Serum amylase POD1 measured**  n =10 (4%) | |  |
|  | **Median/N IQR/%** | | | **Median/N IQR/%** | | **P-Value** |
| CDC-Classification |  | |  |  |  | 0.292 |
| 0 | 33 | 19 % | | 4 | 40 % |  |
| 1 | 10 | 6 % | | 0 | 0 % |  |
| 2 | 55 | 32 % | | 2 | 20 % |  |
| 3a | 26 | 15 % | | 1 | 10 % |  |
| 3b  4a  4b | 22  17  3 | 13 %  10 %  2 % | | 0  3  0 | 0 %  30 %  0 % |  |
| 5 | 6 | 4 % | | 0 | 0 % |  |
| Intraabdominal abscess | 26 | 15 % | | 2 | 20 % | 0.677 |
| Bile duct anastomosis leak | 14 | 8 % | | 0 | 0 % | 0.348 |
| Gastroenterostomy leak | 8 | 5 % | | 0 | 0 % | 0.485 |
| Burst abdomen | 9 | 5 % | | 1 | 10 % | 0.520 |
| SSI with wound reopening | 32 | 19 % | | 1 | 10 % | 0.492 |
| POPF |  |  | |  |  | 0.211 |
| None/ POPF A | 146 | 85 % | | 7 | 70 % |  |
| POPF B/C | 26 | 15 % | | 3 | 30 % |  |
| DGE |  |  | |  |  | 0.543 |
| None/ DGE A | 142 | 83 % | | 9 | 90 % |  |
| DGE B/C | 30 | 17 % | | 1 | 10 % |  |
| PPH |  |  | |  |  | 0.944 |
| None/ PPH A/B | 136 | 79 % | | 8 | 80 % |  |
| PPH C | 36 | 21 % | | 2 | 20 % |  |
| PPH intraluminal | 22 | 13 % | | 2 | 20 % | 0.512 |
| PPH extraluminal | 11 | 6 % | | 0 | 0 % | 0.409 |
| Unplanned ventilation | 17 | 10 % | | 2 | 20 % | 0.309 |
| Postoperative hemodialysis | 5 | 3 % | | 0 | 0 % | 0.585 |
| Pneumonia | 35 | 20 % | | 5 | 50 % | **0.028** |
| Discharge circumstance |  |  | |  |  | 0.646 |
| Home | 136 | 79 % | | 9 | 90 % |  |
| Hospice/ death | 7 | 4 % | | 0 | 0 % |  |
| Hospital | 12 | 7 % | | 1 | 10 % |  |
| Rehabilitation | 17 | 10 % | | 0 | 0 % |  |
| Reoperation | 51 | 30 % | | 4 | 40 % | 0.754 |
| Histology risk group |  |  | |  |  | 0.189 |
| Yes | 80 | 47 % | | 2 | 20 % |  |
| No | 92 | 53% | | 8 | 80 % |  |
| Readmission | 24 | 14 % | | 2 | 20 % | 0.595 |
| ICU stay days | 1 | 0 – 79 | | 2 | 1 – 30 | 0.660 |
| IHM | 6 | 4 % | | 0 | 0 % | 0.548 |

| **Table 3. Laboratory values on postoperative day one of patients with serum amylase or not** | | | | | | | |  |
| --- | --- | --- | --- | --- | --- | --- | --- | --- |
|  | | **Serum amylase**  **not measured POD 1**  n=172 (95%) | | | **Serum amylase measured on POD1**  n =10 (5%) | |  |  |
|  | | **Median/N IQR/ %** | | | **Median/N IQR/ %** | | **P-Value** | **Missing values (n)** |
| **Serum amylase (u/l) day 1** | XX | |  | | 147 | (5 - 7500) | XX | 172 & 0 |
| **Drain amylase (u/l) day 1** | 277 | | (3 - 73614) | | 109 | (28 - 5536) | 0.920 | 10 & 1 |
| **Serum lipase (u/l) day 1** | 88 | | (3 - 1614) | | 145 | (6 - 1054) | 0.924 | 3 & 2 |
| **Drain lipase (u/l) day 1** | 793 | | (4 - 21900) | | 10185 | (65 - 31662) | 0.183 | 60 & 6 |
| **CRP (mg/L) day 1** | 79 | | (6 - 306) | | 96 | (80 - 258) | 0.086 | 109 & 4 |
| **WBC (n/L) day 1** | 12280 | | (5730 - 32240) | | 12655 | (9550 - 19930) | 0.541 | 2 & 0 |
| **Urea (mmol/L) day 1** | 4.95 | | (2.38 - 14.48) | | 8.76 | (8.76 – 8.76) | 0.248 | 134 & 1 |
|  | |  | |  |  |  |  |  |
